# *Cryptotermes colombianus* a new drywood termite and distribution record of Cryptotermes in Colombia

**DOI:** 10.3897/zookeys.596.9080

**Published:** 2016-06-07

**Authors:** Robin Casalla, Rudolf Scheffrahn, Judith Korb

**Affiliations:** 1 Universität Freiburg. Evolutionary Biology & Ecology. Hauptstrasse 1. Freiburg 79104. Germany; 2 Universidad del Norte. Departamento de Química y Biología. Kilómetro 5 Antigua vía Puerto Colombia. Barranquilla. Colombia; 3 University of Florida. Fort Lauderdale Research & Education Center 3205 College Avenue Davie. Florida 33314. United States

**Keywords:** Cryptotermes colombianus new species, Cryptotermes cylindroceps, Cryptotermes mangoldi, tropical dry forest, Colombian Caribbean coast

## Abstract

A new species of drywood termite (Kalotermitidae), *Cryptotermes colombianus*, is described and new records for *Cryptotermes cylindroceps and Cryptotermes mangoldi* are presented from the Caribbean coast of Colombia. *Cryptotermes colombianus* is described from two soldiers and genetic sequences. This unusual species differs noticeably from other regional *Cryptotermes* species for its weak and inconspicuous definition of the frontal and genal horns and its acute angle of the frons with respect to the vertex. *Cryptotermes colombianus* clustered with species from the Ethiopian and Oriental region and it is closely related to *Cryptotermes havilandi*. *Cryptotermes cylindroceps* is widely distributed along the Colombian Caribbean coast, commonly associated with dead wood in mangrove habitats. It also is commonly found in wooden furniture, constituting an important household pest. *Cryptotermes mangoldi* is reported from the Caribbean mainland for the first time.

With these new records, there are now five *Cryptotermes* species for Colombia, including the pest species *Cryptotermes brevis* and *Cryptotermes dudleyi*. This new description raises the numbers of Neotropical *Cryptotermes* to a total of 34 species, of which 2 are fossils, 4 introduced, and 28 endemic.

## Introduction

*Cryptotermes* is one of the best studied and economically most significant genus of drywood termites (Krishna 1961, Chhotani 1970, Gay and Watson 1982, Lenz et al. 1985, Bacchus 1987, Constantino 1998, Scheffrahn and Křeček 1999, Korb 2009, Krishna et al. 2013). Sixty-nine species have been described with 33 distributed in the Neotropics (2 fossil, 4 introduced and 27 native species, including *Cryptotermes venezolanus* - *nomen dubium*).

*Cryptotermes* has been poorly studied in Colombia, only three species have been recorded: *Cryptotermes brevis* (Walker 1853), *Cryptotermes dudleyi* Banks 1918, and *Cryptotermes cylindroceps* Scheffrahn and Křeček 1999, (Gile et al. 2011). The first two species are important pests. *Cryptotermes dudleyi* has been introduced to the Neotropics and often appears in disturbed outdoor habitats (Scheffrahn and Křeček 1999, Constantino 2002) and *Cryptotermes brevis* whose origin was established for the Atacama Desert region of coastal northern Chile and southern Peru is widespread in the Neotropics, (Scheffrahn et al. 2009). Until this study *Cryptotermes mangoldi* Scheffrahn and Křeček 1999, was only known from the Dominican Republic.

Morphological identification of termite species can be difficult as diagnostic morphological markers can be rare and are often restricted to soldiers or alates. For such taxa, sequencing of gene fragments (DNAbarcoding) is now an important molecular tool widely used to elucidate phylogenetic relationships between taxa and to identify species (Inward et al. 2007a, 2007b, Legendre et al. 2008). Mitochondrial markers have been extensively used in termites, e.g. Miura et al. 2000, Lo et al. 2004, Ohkuma et al. 2004, Bergamaschi et al. 2007, Li et al. 2009, Singla et al. 2013, Hausberger et al. 2011, Scheffrahn et al. 2015. In termites, sequencing fragments of the cytochrome oxidase subunit II (COII) proved to be an especially suitable marker (e.g., Legendre et al. 2008, Hausberger et al. 2011): Cytochrome oxidase subunit I (COI), the standard ‘tree of life gene’, is less suitable as it does not amplify well in termites and have too low resolution to distinguish species.

Most studies on Colombian termites have been directed towards species of economic importance as pests in agriculture and forestry (Weidner 1980, Galvis 1985, Scheffrahn et al. 1999, Scheffrahn 2010, 2011, Gutierrez et al. 2004, Medina and Pinzon-Florian 2011, Abadía et al. 2013). The total number of termite species in Colombia remains unknown, but Vargas-Niño et al. (2005) list 26 genera of Termitidae from Colombia. Given that Colombia has 37 types of ecosystems (Instituto de Hidrología, Meteorología y Estudios Ambientales et al. 2007) and more than 26,000 plants (Bernal et al. 2015), 2,569 from tropical dry forests (Instituto de Investigación Alexander von Humboldt 2014), the number of termite species for Colombia is expected to be high.

The purpose of this paper is to describe a new *Cryptotermes* species, *Cryptotermes colombianus*, and to provide new information on the status, biology and distribution of genus *Cryptotermes* in Colombia.

## Materials and methods

Specimens were gathered as part of a research project on termite assemblages in the Colombian Caribbean between 2014 and 2015. Termites were collected using a standardized sampling protocol (Jones and Eggleton 2000, Hausberger and Korb 2015). Termites were also collected in structural wood from buildings and furniture. All *Cryptotermes* were preserved in 100% ethanol for DNA analysis, and 80% ethanol for museum curation. Additional *Cryptotermes* localities from Colombia are included in this paper, from an unpublished survey in 2009 by R. Scheffrahn.

### Identification

Taxonomic keys from Scheffrahn and Křeček (1999) were used to determine *Cryptotermes* species. The specimens of the new species could not be identified with this key. Hence, it was sequenced together with specimens from all samples, except *Cryptotermes mangoldi*, for genetic species identification. In addition, eleven other *Cryptotermes* species and *Blatta orientalis* were used for comparison (Table 1). Fragments of the mitochondrial gene *cytochrome oxidase subunit II* (*COII*; total length ~740 bp), 12S rRNA (~385 bp) and 16S rRNA (total length ~480bp) were used and sequenced as described in Hausberger et al. (2011). DNA sequences were aligned with MEGA 6.0. (Tamura et al. 2013) and a Bayesian inference phylogeny was created with MrBayes 3.2.1. (Ronquist and Huelsenbeck 2003) (10^7^ generations, 50% discarded as burn-in). The resultant tree was visualized using FigTree version 1.4.2 (http://tree.bio.ed.ac.uk/software/figtree/). Additionally, we also used MEGA 6.0. to calculate *p*-distance between species.

**Table 1.** GenBank accession numbers of the mitochondrial genes.

Species	GenBank ID		
	COII	12S rRNA	16S rRNA
*Blatta orientalis*	DQ874267.1	-	-
*Cryptotermes cavifrons*	FN377806.1	-	-
*Cryptotermes colombianus*	KU510330	KX267100	KX267099
*Cryptotermes cylindroceps*	KU510331	-	-
*Cryptotermes declivis*	HQ012042.1	-	-
*Cryptotermes domesticus*	AF189085.1	-	-
*Cryptotermes dudleyi*	FN377808.1	-	-
*Cryptotermes havilandi*	FN377809.1	-	-
*Cryptotermes longicollis*	FN377810.1	-	-
*Cryptotermes primus*	AF189090.1	-	-
*Cryptotermes queenslandis*	AF189092.1	-	-
*Cryptotermes secundus*	AF189093.1	-	-
*Cryptotermes simulatus*	AF189094.1	-	-
*Cryptotermes tropicalis*	AF189095.1	-	-

### Imaging and measurements

Specimens were suspended in Sagrotan® Hand Sanitizer and images were taken with a Nikon SMZ25 stereomicroscope coupled to a Nikon Model DS-Fi2 digital camera. The software Helicon Focus® was used to stack pictures. Morphological definitions and measurements were done following Roonwal (1969), Gay and Watson (1982) and Scheffrahn and Křeček (1999).

### Deposit

Voucher specimens are held at Freiburg University. The holotype, dealated morphotype and pseudergates from type colony of *Cryptotermes colombianus* will be deposited in the Natural History Museum of the Alexander von Humboldt Institute of Bogotá (MIAvH) and Paratype soldier in the collection of the American Museum of Natural History, New York. Specimens of *Cryptotermes cylindroceps* will be part of the collection of the Department of Chemistry and Biology at the University del Norte, Barranquilla, Colombia. Other Colombian material is housed in the University of Florida Termite Collection in Davie, Florida.

## Systematics

### Family Kalotermitidae Froggatt, 1897 Genus *Cryptotermes* Banks, 1906

#### Cryptotermes colombianus sp. n.

Taxon classificationAnimaliaIsopteraKalotermitidae

http://zoobank.org/9D27B3AE-E8A0-4512-8A1E-D9E54A88A46C

Fig. 1

##### Description.

**Figure 1.**
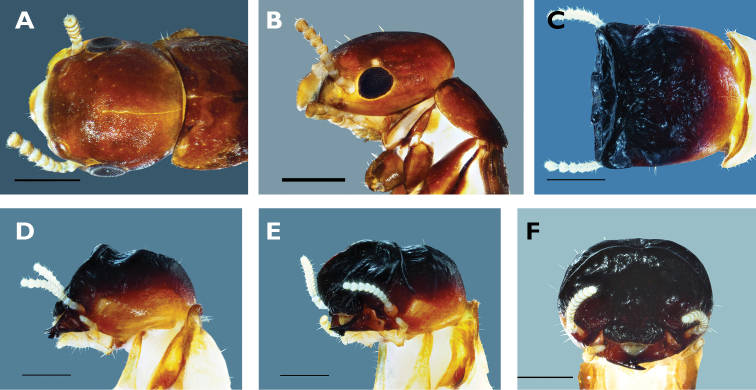
C*ryptotermes colombianus* sp. n. Dealated imago: Head in dorsal (**A**) and lateral view (**B**). Soldier: Head in dorsal (**C**), lateral (**D**), oblique (**E**), and frontal view (**F**). Scale bar: 0.5 mm.

**Dealated** (Fig. 1A–B). General color brown. Frons pale brown, vertex brown. Pronotum and abdominal tergites brown. Antennae pale brown. Labrum pale brown. Femora brown, tibiae pale brown. Abdominal sternites pale brown and very pale brown laterally. Head suboval; cranial sutures fine, but distinct. Eyes moderately large, non-protruding, and oval. Ocelli moderately large, oval, and touching eyes. Antenna with 6 and 8 articles but incomplete, with formulae 2>3<4=5=6. Pronotum wider than long, usually with distinctive midline mark. Arolia present. Measurements are reported in Table 2.

**Table 2.** Measurements (in mm) of *Cryptotermes colombianus* sp. n. dealated imago.

No.	Measurements in mm (n=1) from 1 colony	
1	Head length with labrum	1.27
2	Head length to postclypeus	1.08
3	Head width, maximum at eyes	0.86
4	Eye diameter, maximun	0.30
5	Eye to head base, minimum	0.16
6	Ocellus diameter	0.08
7	Pronotum, maximum width	0.90
8	Pronotum, maximum length	0.73
9	Total length without wings	4.60
10	Total length with wings	–
11	Fore wing length to suture	–
12	Fore wing, maximun width	–

**Soldier.** (Fig. 1C–F). Head in dorsal view with frontal flange and front horns very dark; 3/4 of anterior vertex almost black chestnut, grading to chestnut brown; posterior it turns ferruginous orange to pale yellow (Figure 1C). Head in lateral view with anterodorsal region almost black, which grades steeply to chestnut brown then to pale yellow under eye spot and occipital foramen (Figure 1D). Mandibles chestnut brown. Anterior margin of pronotum chestnut brown posterior margin pale yellow (Fig. 1E–F).

Head in dorsal view abruptly truncated in front; frontal flange forming a rim surrounding a few undulations on frons. Head widest behind flange, gradually narrowing toward the occiput (Figure 1C). Frontal flange coalesces with frontal horn and postclypeus to form pentagonal rim occupying the entire frontal view. In lateral view, margin of frons and occiput from acute ca. 60 degree angle (Fig. 1D–E). Vertex widely striated with several robust undulations; frontal horns very broad and shallow; genal horns reduced to tiny protrusions anterior to antennal sockets. Mandibles short humped and slightly bended forward, right mandible tip under tip of left mandible, tips are under labrum in frontal view. Labrum short, hyaline and tongue-shaped. Anteclypeus white; postclypeus trapezoidal with undulating rugosity. Eye spots large, narrowly elliptical. Antenna moniliform between 10 and 12 articles, formula variable 2> 3 = 4 = 5 <6. Legs with three apical spurs on each tibia, formula 3:3:3. Pronotum slightly incised in front, slightly narrower than head capsule. Measurements are reported in Table 4.

##### Genetic characterization.

Thirteen COII mtDNA sequences were aligned for *Cryptotermes* species using *Blatta orientalis* as an outgroup. Information from NCBI is largely limited to COII (see Suppl. material 1), hence we could not include comparative analysis for nuclear and mitochondrial 12S and 16S rRNA genes. Note, COII is very informative to identify termite species (Hausberger et al. 2011).

**Figure 2.**
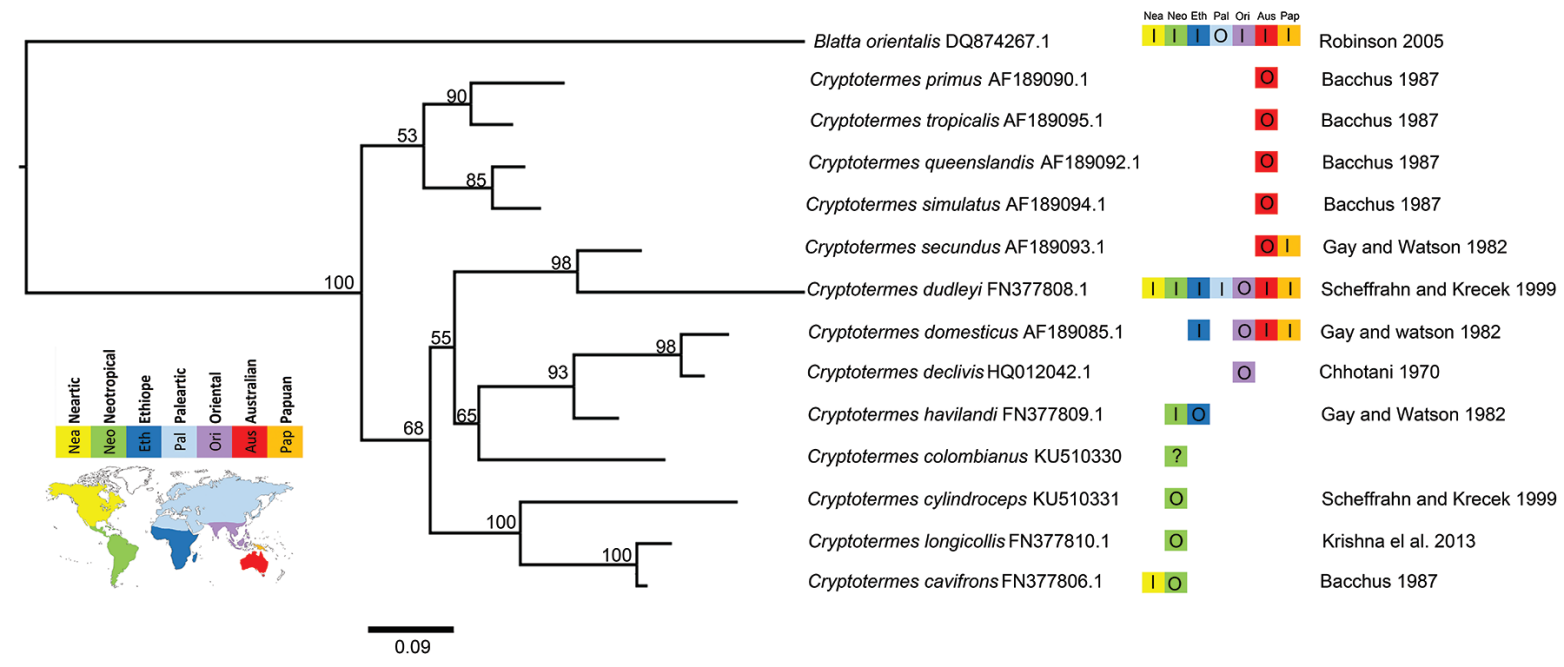
Tree topology and branch lengths inferred with MRBAYES from COII sequence data (Bootstrap values above branches). Origin (O), unknown (?) and established introductions from other regions or land masses (I): Neartic= Nea, Neotropic= Neo, Ethiopian= Eth, Paleartic= Pal, Oriental= Ori, Australian= Aus, Papuan= Pap.

The COII tree topology for *Cryptotermes* revealed two major clusters, one group composed of eastern Australian species (53% bootstrap value) and the other comprising clusters of Northwest Australian-Papuan (98% bootstrap value), Ethiopian-Oriental (65% bootstrap value) and Neotropical species (100% bootstrap value) (Figure 2). *Cryptotermes colombianus* is located on a separate basal branch within the Ethiopian–Oriental cluster. Based on additional sequence comparisons, its closest relative among the studied species is *Cryptotermes havilandi* (p-distance = 0.148) (Table 3).

**Table 3.** Estimates of Evolutionary Divergence between Sequences (*p*-distance between species).

	Species	1	2	3	4	5	6	7	8	9	10	11	12	13
**1**	*Cryptotermes cavifrons*													
**2**	*Cryptotermes longicollis*	0.030												
**3**	*Cryptotermes cylindroceps*	0.157	0.165											
**4**	*Cryptotermes primus*	0.174	0.186	0.184										
**5**	*Cryptotermes tropicalis*	0.158	0.172	0.167	0.096									
**6**	*Cryptotermes queenslandis*	0.167	0.177	0.162	0.130	0.117								
**7**	*Cryptotermes simulatus*	0.165	0.188	0.160	0.137	0.132	0.064							
**8**	*Cryptotermes secundus*	0.174	0.183	0.179	0.179	0.163	0.153	0.165						
**9**	*Cryptotermes dudleyi*	0.200	0.202	0.188	0.209	0.190	0.188	0.205	0.137					
**10**	*Cryptotermes havilandi*	0.150	0.160	0.167	0.160	0.137	0.151	0.167	0.170	0.183				
**11**	*Cryptotermes domesticus*	0.165	0.172	0.190	0.160	0.146	0.174	0.177	0.188	0.216	0.113			
**12**	*Cryptotermes declivis*	0.169	0.176	0.183	0.167	0.150	0.181	0.177	0.176	0.203	0.108	0.059		
**13**	*Cryptotermes colombianus*	0.183	0.186	0.167	0.172	0.160	0.169	0.162	0.186	0.202	0.148	0.150	0.160	
**14**	*Blatta orientalis*	0.287	0.296	0.257	0.247	0.256	0.256	0.254	0.270	0.285	0.264	0.237	0.249	0.278

**Table 4.** Measurements (in mm) of *Cryptotermes colombianus* sp. n. soldier.

No.	Measurements in mm, n=2 from 1 colony	(Holotype)	(Paratype)	Mean
1	Head length to tip of mandibles	1.54	1.38	1.46
2	Head length to frontal horns	1.33	1.23	1.28
3	Frontal flange width	1.32	1.22	1.27
4	Frontal horns, outside span	1.32	1.22	1.27
5	Head width, maximum	1.32	1.22	1.27
6	Head height, excluding postmentum	1.01	0.88	0.94
7	Pronotum, maximum width	1.16	1.14	1.15
8	Pronotum, maximum length	0.82	0.77	0.79
9	Left mandible length, tip to ventral condyle	–	–	–
10	Total length	4.18	3.95	4.07

Phylogeny and phylogeography of the *Cryptotermes* is debated (Chhotani 1970, Gay and Watson 1982, Bacchus 1987, Thompson et al. 2000, Scheffrahn and Křeček 2009). Bourguignon et al. (2014) proposed that Kalotermitidae evolved at the cusp of Gondwana dissolution with *Cryptotermes* originating after the separation of land masses. The current distribution of *Cryptotermes* species can be explained with transoceanic dispersal via drift wood (Scheffrahn et al. 2009, Bourguignon et al. 2016) and more recently through human introductions during colonization and trade (Li et al. 2009, Scheffrahn et al. 2009, Evans 2011). The geographic pattern on the phylogeny with regional specific clades may also indicative for some continent specific radiations. The origin of *Cryptotermes colombianus* is unclear, it may have arrived in Colombia via infested drift wood. Data presented here are not conclusive. More genetic analyses, including different populations, are needed to reveal the origin of *Cryptotermes colombianus* and track the evolutionary history and dispersal of *Cryptotermes* species.

##### Material examined.

Type-locality: Colombia, Magdalena: Santa Marta, Tayrona National Park, Gayraca Bay, 11°18.84'N; 74°6.34'W, tropical dry forest, 23 June 2015.

Holotype-colony: Colombia. Magdalena Santa Marta Tayrona National Park, Gayraca Bay, 23.VI.2015 (collected by R. Casalla) in a piece of dry wood on soil, at elevation of 12 m a.s.l (11°18.84'N; 74°6.34'W), sample COLPT1LII-56: 2 soldiers, 1 dealated, 23 pseudergates; 3 for DNA isolation. Holotype: Soldier from the previous sample (COLPT1LII-56), it will be deposited at the Arthropod Collection of the Natural History Museum of the Alexander von Humboldt Institute of Bogotá, Colombia (MIAvH). Paratypes from sample COLPT1LII-56: 1 soldier, 1 reproductive dealate. Paratypes will be deposited as follows: 1 soldier will be deposited at the American Museum of Natural History New York, United States, 1 dealated at MIAvH. Pseudergates will be part of the collection of the Department of Chemistry and Biology at the University del Norte, Barranquilla, Colombia. All measurements for dealated reproductive, holotype and paratype soldiers are reported in Tables 2, 4.

##### Diagnosis.

The diminutive frontal and genal horns and the truncated frons and converging genal margins of the head capsule (in dorsal view) distinguish the *Cryptotermes colombianus* soldier from all other Neotropical congeners.

##### Etymology.

Named for its country of origin, Colombia.

## Discussion

**Figure 3.**
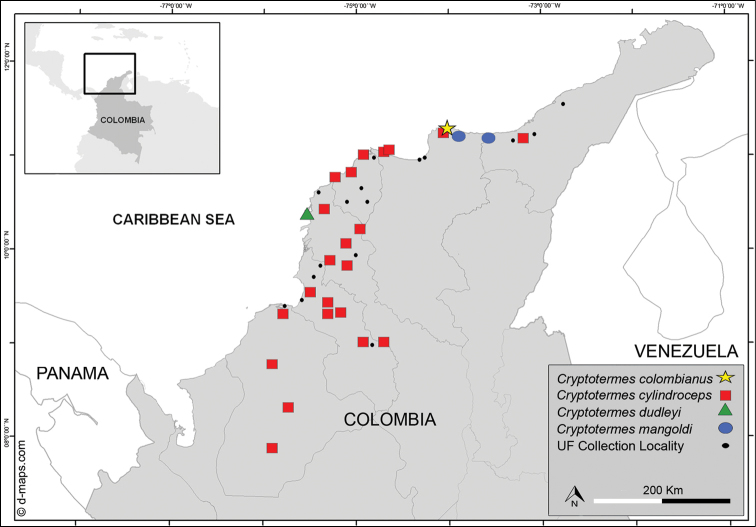
Distribution of the genus *Cryptotermes* in Colombia. *Cryptotermes brevis* not shown, but widespread.

**Figure 4.**
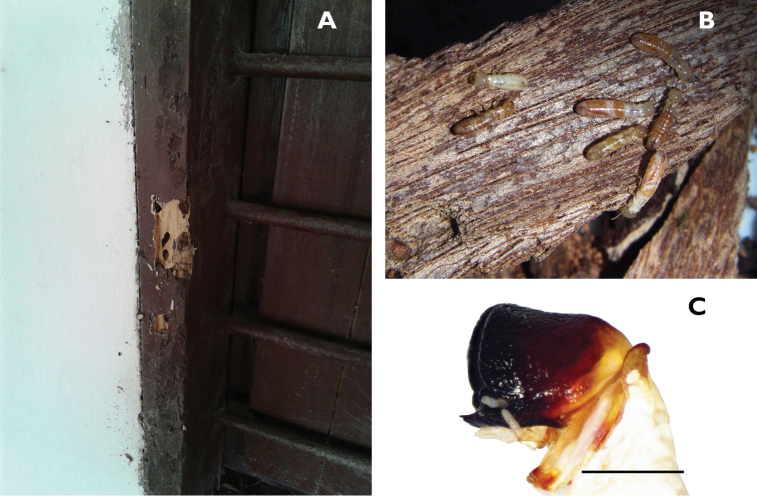
Window frame damaged by *Cryptotermes cylindroceps* (**A**), workers (white-reddish) (pseudergates *sensu lato*) and neotenic reproductives (brownish) (**B**), soldier of *Cryptotermes cylindroceps* (**C**). Scale bar: 1 mm

We extend the distribution of *Crytotermes* to Colombia and we herein report *Cryptotermes mangoldi* for the first time, along the Caribbean coast (Figure 3). We found *Cryptotermes cylindroceps* in infested drywood trunks of *Glírícidia sepium*, *Prosopis juliflora*, *Manilkara zapota*, *Hura crepitans* and *Avicennia germinalis*, which are often used for wooden artefacts, furniture and as structural material (Figure 4). Along the coast, *Cryptotermes cylindroceps* was common in dead branches and trunks of the black mangrove, *Avicennia germinalis*. In line transects that covered a total of 500m × 2m, *Cryptotermes cylindroceps* accounted for 24 % of all termite encounters (N = 241) (Casalla and Korb, unpublished data). *Cryptotermes cylindroceps* also occurred up to 100 km inland (Figure 3). Hence, *Cryptotermes cylindroceps* can be considered an economically important pest to this part of the Caribbean. *Cryptotermes mangoldi* was only known from the Dominican Republic (Scheffrahn and Křeček 1999). In 2009, R. Scheffrahn found three samples from two localities near Santa Marta, Colombia (Figure 4).

Genetically, *Cryptotermes cylindroceps* clustered with the other Neotropical endemics, *Cryptotermes cavifrons* and *Cryptotermes longicollis* (100% bootstrap value) (Figure 2). Our data provided strong branch support at the regional level, but more resolution from different species are needed to attain a well-corroborated phylogeny of the *Cryptotermes*.

With these new records, there are now five *Cryptotermes* species recorded for Colombia: *Cryptotermes brevis*, *Cryptotermes colombianus*, *Cryptotermes cylindroceps*, *Cryptotermes dudleyi* and *Cryptotermes mangoldi*. Further studies on the diversity of termites will determine if there are more *Cryptotermes* in northern and western Colombia, especially at the pacific coast which has important mangroves areas.

## Supplementary Material

XML Treatment for Cryptotermes colombianus

## Supplementary materials

Supplementary material 1 List of *Cryptotermes* of the world and sequences reported in the NCBI for mtDNA genes Data type: Data table Excel Explanation note: Hits for mtDNA sequences to COII, 12S, 16S and CytB in *Cryptotermes*. NCBI Filter: ("*Cryptotermes*"[Organism] OR cryptotermes[All Fields]) AND (animals[filter] AND biomol_genomic[PROP] AND mitochondrion[filter]). Updated 05.05.16. This dataset is made available under the Open Database License (http://opendatacommons.org/licenses/odbl/1.0/). The Open Database License (ODbL) is a license agreement intended to allow users to freely share, modify, and use this Dataset while maintaining this same freedom for others, provided that the original source and author(s) are credited.Robin Casalla, Rudolf Scheffrahn, Judith Korb
